# Supplementary material for: Comparative analysis of transposed element insertion within human and mouse genomes reveals Alu's unique role in shaping the human transcriptome
Source: Genome Biol. 2007 Jun 27;8(6):R127. doi: 10.1186/gb-2007-8-6-r127 (PMC2394776; doi:10.1186/gb-2007-8-6-r127)
Supplement: Additional data file 1 — Presented is a table describing the number of exonizations based on the existence of two ESTs/cDNA. [file gb-2007-8-6-r127-S1.doc]

**Table S1**: **The number of exonizations based on existence of at least two ESTs/cDNAs, confirming their appearance.**

**Human**

| **TE** | **Total** | **intronic** | **TE in introns of UCSC annotated genes** | **TE in introns of non-annotated genes** | **TE Exonization in UCSC annotated genes** | **TE Exonization in non-annotated genes** |
| --- | --- | --- | --- | --- | --- | --- |
| *Alu* | 1,094,409 | 718,460 | 480,052 | 238,408 | 432 (0.09%) | 174 (0.07%) |
| MIR | 537,730 | 351,366 | 231,893 | 119,473 | 86 (0.04%) | 34 (0.03%) |
| L1 | 830,062 | 486,901 | 282,146 | 204,755 | 93 (0.03%) | 89 (0.04%) |
| L2 | 375,116 | 240,350 | 154,309 | 86,041 | 44 (0.03%) | 26 (0.03%) |
| CR1 | 50,156 | 33,365 | 22,087 | 11,278 | 4 (0.02%) | 3 (0.02%) |
| LTR | 654,897 | 292,456 | 136,461 | 155,995 | 72 (0.05%) | 145 (0.09%) |
| DNA | 389,688 | 226,489 | 145,968 | 80,521 | 46 (0.03%) | 49 (0.06%) |
| Total | 3,932,058 | 2,349,387 | 1,452,916 | 896,471 | 777 (0.05%) | 520 (0.05%) |

**Mouse**

| **TE** | **Total** | **intronic** | **TE in introns of UCSC annotated genes** | **TE in introns of non-annotated genes** | **TE Exonization in UCSC annotated genes** | **TE Exonization in non-annotated genes** |
| --- | --- | --- | --- | --- | --- | --- |
| B1 | 506,528 | 331,015 | 189,268 | 141,747 | 60 (0.03%) | 42 (0.03%) |
| MIR | 116,355 | 66,597 | 41,853 | 24,744 | 20 (0.05%) | 7 (0.03%) |
| B2 | 338,642 | 215,264 | 118,646 | 96,618 | 55 (0.05%) | 34 (0.04%) |
| B4 | 345,646 | 216,550 | 119,827 | 96,723 | 24 (0.02%) | 23 (0.02%) |
| ID | 45,955 | 30,285 | 18,022 | 12,263 | 4 (0.02%) | 1 (<0.01%) |
| L1 | 820,434 | 457,705 | 181,292 | 276,413 | 39 (0.02%) | 59 (0.02%) |
| L2 | 56,518 | 34,923 | 18,963 | 15,960 | 9 (0.05%) | 3 (0.02%) |
| CR1 | 11,812 | 7,167 | 3,779 | 3,388 | 0 (0%) | 0 (0%) |
| LTR | 756,324 | 396,226 | 156,690 | 239,536 | 55 (0.03%) | 65 (0.02%) |
| DNA | 124,202 | 75,200 | 40,428 | 34,772 | 5 (0.01%) | 1 (0.002%) |
| Total | 3,122,416 | 1,830,932 | 888,768 | 942,164 | 271 (0.03%) | 192 (0.02%) |
