# Supplementary material for: Comparative analysis of transposed element insertion within human and mouse genomes reveals Alu's unique role in shaping the human transcriptome
Source: Genome Biol. 2007 Jun 27;8(6):R127. doi: 10.1186/gb-2007-8-6-r127 (PMC2394776; doi:10.1186/gb-2007-8-6-r127)
Supplement: Additional data file 2 — Presented is a table of all χ2 test P values of all TE exonization levels. [file gb-2007-8-6-r127-S2.doc]

**Table S2: Chi-square test value of exonizations level**

| **Chi-square test between the exonization level of the following retroelements** | **Degree of freedom** | **p-value** |
| --- | --- | --- |
| Alu, MIR (human), L1 (human), L2 (human), CR1 (human) | 4 | <10^-16^ |
| MIR (human), L1 (human), L2 (human), CR1 (human), DNA (human) | 4 | 0.26 |
| MIR (human), L1 (human), L2 (human), CR1 (human), DNA (human), LTR (human) | 5 | 0.00001 |
| B1, MIR (mouse), B2, B4, ID, L1 (mouse), L2 (mouse), CR1 (mouse) | 7 | 0.18 |
| B1, MIR (mouse), B2, B4, ID, L1 (mouse), L2 (mouse), CR1 (mouse), LTR(mouse) | 8 | 0.047 |
| B1, MIR (mouse), B2, B4, ID, L1 (mouse), L2 (mouse), CR1 (mouse), DNA(mouse) | 8 | 0.022 |
| B1, MIR (mouse), B2, B4, ID, L1 (mouse), L2 (mouse), CR1 (mouse), LTR (mouse), DNA(mouse) | 9 | 0.007 |
| MIR (human), MIR (mouse) | 1 | 0.38 |
| L2 (human), L2 (mouse) | 1 | 0.44 |
| CR1 (human), CR1 (mouse) | 1 | 0.23 |
| L1 (human), L1 (mouse) | 1 | 0.007 |
| LTR (human), LTR (mouse) | 1 | <10^-11^ |
| DNA (human), DNA (mouse) | 1 | 0.004 |
