# Supplementary material for: Comparative analysis of transposed element insertion within human and mouse genomes reveals Alu's unique role in shaping the human transcriptome
Source: Genome Biol. 2007 Jun 27;8(6):R127. doi: 10.1186/gb-2007-8-6-r127 (PMC2394776; doi:10.1186/gb-2007-8-6-r127)
Supplement: Additional data file 3 — Presented is a table of statistical χ2 test P values for the preference of all TE exonizations in the sense/antisense orientation. [file gb-2007-8-6-r127-S3.doc]

**Table S3: chi-square test values for calculating biased sense/antisense exonization orientation**

Human Chi-test biased exonization orientation

| **TE** | **χ^2^ test p-value** | **Degree of freedom** | **sense/antisense** |
| --- | --- | --- | --- |
| Alu | p-value < 10^-109^ | 1 | antisense |
| MIR | p-value < 0.00001 | 1 | antisense |
| L1 | p-value = 0.064 | 1 | No bias |
| L2 | p-value = 0.6 | 1 | No bias |
| CR1 | p-value= 0.26 | 1 | No bias |
| LTR | p-value < 0.001 | 1 | sense |
| DNA | p-value= 0.67 | 1 | No bias |

Mouse chi-test biased exonization orientation

| **TE** | **χ^2^ test p-value** | **Degree of freedom** | **sense/antisense** |
| --- | --- | --- | --- |
| B1 | p-value < 10^-6^ | Degree of freedom | antisense |
| B2 | p-value < 0.01 | 1 | antisense |
| B4 | p-value < 0.02 | 1 | antisense |
| MIR | p-value < 0.001 | 1 | antisense |
| L1 | p-value = 0.1 | 1 | No bias |
| LTR | p-value = 0.02 | 1 | sense |
| DNA | p-value = 0.76 | 1 | No bias |
