# Supplementary material for: Comparative analysis of transposed element insertion within human and mouse genomes reveals Alu's unique role in shaping the human transcriptome
Source: Genome Biol. 2007 Jun 27;8(6):R127. doi: 10.1186/gb-2007-8-6-r127 (PMC2394776; doi:10.1186/gb-2007-8-6-r127)
Supplement: Additional data file 4 — Presented 4 is a table of statistical χ2 test P values for the preference of all TE exonizations in the UTR. [file gb-2007-8-6-r127-S4.doc]

**Table S4**: **Statistical significance calculation of χ^2^ test p-value for UTR preference.** The null hypothesis checked is 21% within the UTR versus 79% CDS for human genes and 27% within the UTR versus 73% in the CDS in mouse genes (see also Materials and Methods).

**Human**

| **RE** | **Number within CDS** | **Number within UTR** | **χ^2^ test p-value** | **Degree of freedom** |
| --- | --- | --- | --- | --- |
| *Alu* | 650 | 396 | < 10^-16^ | 1 |
| MIR | 100 | 78 | <1.3*10^-13^ | 1 |
| L1 | 144 | 71 | <2*10^-5^ | 1 |
| L2 | 66 | 35 | <8.4*10^-4^ | 1 |
| LTR | 76 | 70 | <2.3*10^-6^ | 1 |
| DNA | 77 | 18 | 0.85 | 1 |

**Mouse**

| **RE** | **Number within CDS** | **Number within UTR** | **χ^2^ test p-value** | **Degree of freedom** |
| --- | --- | --- | --- | --- |
| B1 | 87 | 43 | < 10^-16^ | 1 |
| MIR | 9 | 15 | < 5*10^-7^ | 1 |
| B2 | 37 | 40 | <3* 10^-11^ | 1 |
| B4 | 39 | 20 | < 0.015 | 1 |
| L1 | 54 | 45 | <2.4*10^-9^ | 1 |
| L2 | 3 | 6 | < 0.0009 | 1 |
| LTR | 38 | 31 | 0.05 | 1 |
