# Supplementary figures and images for: Comparative analysis of transposed element insertion within human and mouse genomes reveals Alu's unique role in shaping the human transcriptome
Source: Genome Biol. 2007 Jun 27;8(6):R127. doi: 10.1186/gb-2007-8-6-r127 (PMC2394776; doi:10.1186/gb-2007-8-6-r127)

**Figure S1: an example of Alu exonization within non-coding gene**

**
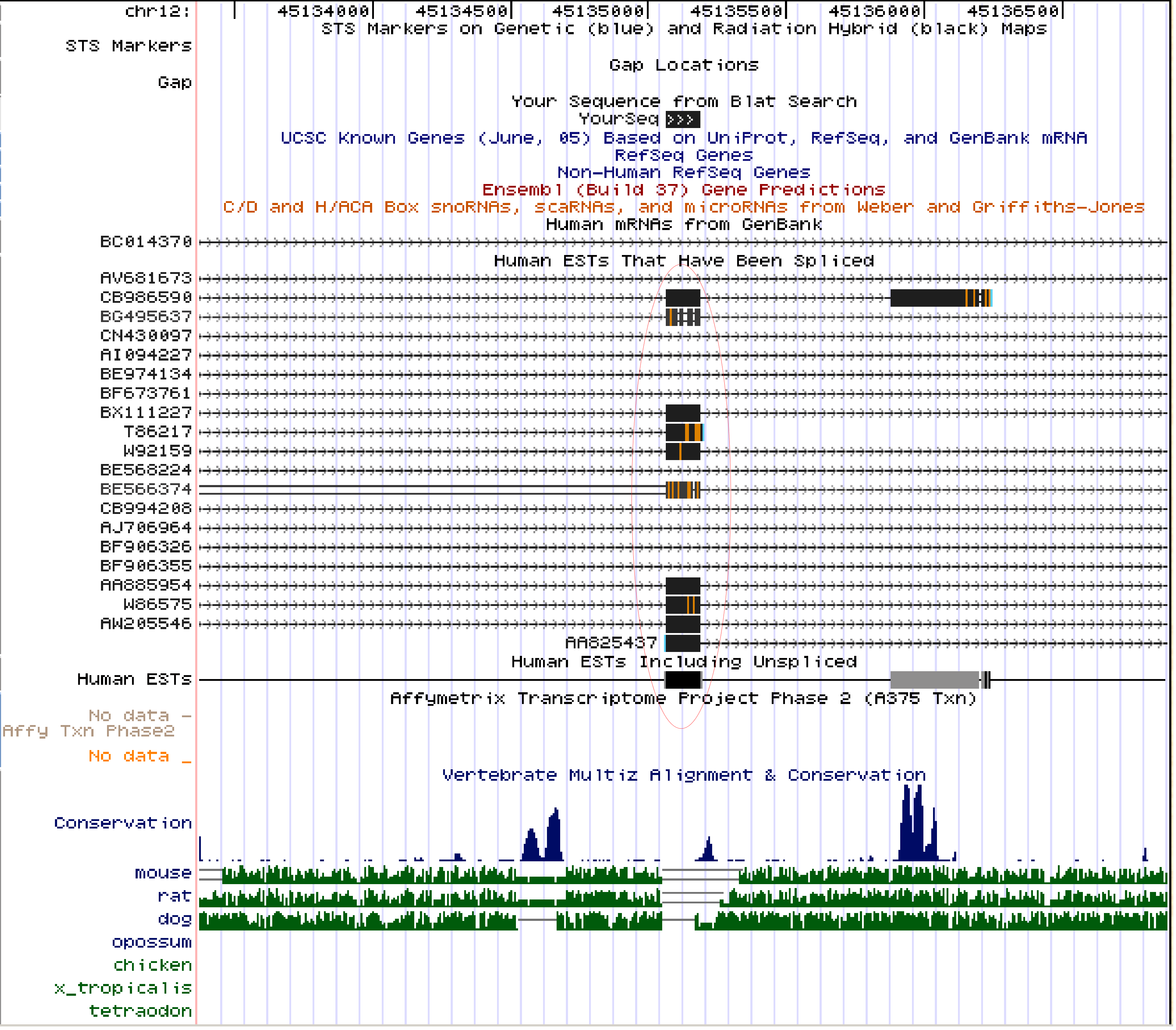
**

Supplement: Additional data file 5 — Presented is an example of Alu exonization within a non-protein-coding gene. [file gb-2007-8-6-r127-S5.doc]
