# Supplementary material for: Comparative analysis of transposed element insertion within human and mouse genomes reveals Alu's unique role in shaping the human transcriptome
Source: Genome Biol. 2007 Jun 27;8(6):R127. doi: 10.1186/gb-2007-8-6-r127 (PMC2394776; doi:10.1186/gb-2007-8-6-r127)
Supplement: Additional data file 6 — Presented is an illustration showing alignment of mouse L1 (Lx8) and human L1 (L1MC4). [file gb-2007-8-6-r127-S6.doc]

**Figure S2:** **Mouse abundant Lx8 versus L1MC4 most abundant within the human genome:**

CLUSTAL W (1.83) multiple sequence alignment

Lx8 -----------------------------------------------------AACACAA

L1MC4 CTAATATACCTAATATACAAAAAACTCTTAAAATTGAAGGATAAAAAGNCAAAAACCNAA

*** **

Lx8 GAGAAAAA-GAAGAAAGAAGATAAATA------CCACTAAGGATNTTCGAAANGCTCTAA

L1MC4 TANNAAAATGGGNAAAAGACATGAACAGACAATTCACNAAAAATNATAAAATGGCCCTTA

* **** * *** * ** ** * *** ** *** * ** ** ** *

Lx8 GGAATC--ATATTAT-TTTATKTTTACCTGAAATGTGTG---TGTGTGTTAAT-TTAAAT

L1MC4 AGCATATAAAAAGATGTTCANCCTCACNTATAATTAGAGAAACGCAAATTAAAACTACAC

* ** * * ** ** * * ** * *** * * * **** ** *

Lx8 GAAGTTATC---------CCACTTGGGS------------------TGATAATGCTCCCT

L1MC4 CGAGATACCATTTCTCACCCANCAGATCGGCAAAAATTAAAAAGTATGGCAATATANNCT

** ** * *** * ** *** **

Lx8 CCAAG---AGCCAAAGACCATCTAACAAAANCCCMAACACCAGGCATGAGAA-RCCCTCT

L1MC4 GTTGGCGAGGCTGTGGGGNAACNGGNACTCTCATACACTGCTGGTGGGAGTGCAAATTGG

* ** * * * * * ** * ** *** *

Lx8 TTCGAGTTGTTGGTCAGGGKWKTCCA-AGAGACTCCCAAAACAATATGGGCTATTGCTAT

L1MC4 TACAACTNCTTTGGAAGANAATTTGGCAGTNTCTAATAAAACTACACNTGCNTTTACACT

* * * * ** * ** * ** ** ***** * * ** ** * *

Lx8 TG---CCMTTGGTTGTCCCAGNAGAGGT-------AGAAGGTAAGTCCCTWTTGCT-GAA

L1MC4 TTGACCCATTAGTCCCACTTCTAGAAATTTACCCTANAGAAATACTTCTAACAGNTCAAA

* ** ** ** * *** * * * * * * * * **

Lx8 GACAC-CATGCAC-------TTCGGACACAGGACCCAGAGGMCCCTGAG---CTGGAACT

L1MC4 AATACACATGTACAGGGATGTTCATAGCAGTNTTATTNNTAATNGTAAAANATTGGAAAC

* ** **** ** *** * * * *****

Lx8 GACCTGAAWATCTTCTCCCTGAGGACTAGCTT------TCATGGTAC-----CAGAAGG-

L1MC4 AATCNAAATGTCCATCAGCAGGAGAATGGNTGAATAAACTATGGTNCATCCACACAATGG

* * ** ** * * ** * * * ***** * ** ** *

Lx8 --TGCTATGCAAGCTTCCAAAGGAGAGAGGCAACKAACAGTCCYACCCAGCCGTGATGCC

L1MC4 AATACTATNCAGCTGTAAAAAAGAATGAGGAAGATCTCTGTAATAATGTGGAGNGATTTC

* **** ** * *** ** **** * * ** * * * *** *

Lx8 TATGAACCACATCAAC---GACCAGCATGGCACAATAACCCCAAGGGTGCAGTAGTGGCA

L1MC4 GGAACATNNTNTTNAGTTGAAAAAGCNANGCGCAA-AAGAGTATATATANTATGCTACCC

* * * * *** ** *** ** * * * * *

Lx8 CKCACACCTT---GGCGGTAACCAACAGCTCTCTAATTGGACTTAAG-RCCTGCTCAACA

L1MC4 TTCATATAAGAAAGAAGGGGATATGAGAAAATATACATATATCTGCTCATTTGTGCAAAA

** * * ** * * ** * * * ** *** *

Lx8 AGA------GGGAAATCATACTTGGTACTGA-AAACCCGGCTAAATACCCATGGCTGGTG

L1MC4 AGAAACACAGAAAAGATAAANCAGANACTAATGAGATTGGTTACCCACAGGGAANNGGTG

*** * ** * * * *** * * ** ** ** ****

Lx8 ARGTCATGGGCCTTAGAGGAGAACCTACA------ACCGCCACTTTACTAAAC-------

L1MC4 GGAATGGGGAGGAAAGGACGGAAGGAATGGGGGGCAGTGACACTTTTCTGAGTATACCTT

** ** *** * * * ****** ** *

Lx8 -CAGTACAATNCCTAACT--GCATTCTAAATATTTATCCTTATACCCACAGATAGGTGTA

L1MC4 TTTGTATAGTTCTAACTTTTGNAACCATGTTAATGTTTCACATACTCA-AGAAATGAATA

*** * * * * * * * * ** * * * **** ** *** * * **

Lx8 GTCC---TCACCCCTCATCAAGGAAACTTCTCTTTGCAA--CAGACAGAGACCATTACAG

L1MC4 ANTAAAATCAACAAGGATGGGGGANAACTCAAAATGAAATACAAACAGAAACAAATGAAC

*** * ** *** * ** ** ** ** ***** ** * * *

Lx8 AAAACCACAAC-CAATCAAAATGCAGAGTTGTG--GAGCCCAGTCCCAGCGGATACATCT

L1MC4 CWAACTGTATTTCAAATGAATAACATAACCACACTGAAGGGGGTNAGGAAGAAAAGAACT

*** * *** ** ** * ** ** * * * * **

Lx8 A--CAAAACAACTCCTGCACCTAAGGCTCAGGGAACAT---CGCGGAAGAGGGAGCAGAA

L1MC4 AACCCAAGTAACTTTTGAACACAGTATTTTGACTATATGCCCTCAGGCTAAAGACAAAAA

* * ** **** ** ** * * * * ** * * * * ** * **

Lx8 AGATTGTAAG-AGCCAGAGGA------TCAGGGAGTTTGGTGT--GAGACTGTGTCTCCT

L1MC4 GAACTNTAAACAAATATTGAACTCTAGTTAGTAGGCTTATTTTCCGCAGNGGCATGGGTT

* * *** * * * * * ** * ** * * * * * *

Lx8 AGTAATGTCAGAAGCTACACCCATGAGGTCTCACCAACATG--ACTGCCTAAACATGAGC

L1MC4 AGCAAT-TCTGAAACTACTTTCTGTATATTCTAGGACTGAGCAAATAAGTAAATATATTG

** *** ** *** **** * * * * * * * * **** **

Lx8 TGAGCAAGGACGACAGCAATGGACAC-GCTAAAGTGGACAGGGGAAAGCTCACCAAGCCT

L1MC4 NGGATAATGGGAGCCAGGTTTCTCACTGTCGGAGAAGGGAGTTACAAATATGGAAAGGGG

* ** * * * *** * ** * ** ** ***

Lx8 CAACCCTACACAAAGAACTACAG-GCAACTAAGGAAT----GCTGAGAGTGGGAGAA-AT

L1MC4 GAAGACTAGAATGAACCCTGTGGTGTTGGATTGGAATTGGAGGTATCAGTGTGAACTCAT

** *** * * ** * * ***** * * **** ** **

Lx8 AGTCTTCCCCCGGGAAGAGCACATCAATTGGTT-ATCCAATACCAAATGGTCAGCCCTGA

L1MC4 GGTTTTTAATATANATAGATATACAGACAGACAGATATAGAAATAGATATAGATATATAT

** ** * * * * * ** * * * ** * *

Lx8 AAACATAYACAYACAAGTAACATTATACAGAC-TGAGCAGGTTGTATTTATGTATTTANG

L1MC4 GTGTNTGTGTATATGTGTATGTATATACGTACATATATTTCCTAGCTCTGTCCACTGAGA

* * * *** ***** ** * * * * * * * *

Lx8 AA---TAGATRYRYRYRYRYRYRYRYRYRYRYRYRYRYRYRYRYRYRYRYRCAA-TTAAW

L1MC4 GGGCCTAGAAGCAATGACACCCCAGTAGCAATGAGCACACCTA-GCGCCCAGATCTTGGT

**** * **

Lx8 GAAAAAAGAGGCCATGAATTTGAAAAGAAGCAWGGAGGNNT----ATATGGGAGGGTTTG

L1MC4 TTCTAAATACCATTCTCCACTAAAAGGAACCAGGGCTCCTTGGAGAAATGGCTGATTCCA

*** * * *** *** ** ** * * **** * *

Lx8 GAGGGAGGAAAGGGAAGG---GAGAAATGNTGTAATTATATTATAATCTCAAAAAATAAA

L1MC4 GGGCTGGGGCAGGGAAAGTACAAGATGAGCCTGGAACATCTTGTTGTGCCAGAAAGTAAG

* * ** ****** * *** * * ** ** * * ** *** ***

Lx8 A-----------------------------------------------------------

L1MC4 GAAGTGCTCAAAGAATGATGGGGACATGTCAAAAGGACACAGGAGCCAGCTTGAAGGGGC

Lx8 ------------------------------------------------------------

L1MC4 TCCCACTGGCCAAATCTGGGACAATTTGAGCATCAAAATAAATAATGATAGTAATGGATT

Lx8 ------------------------------------------------------------

L1MC4 ATAACCCATTGAATAAAATAAGAATCCATGAGTCCATACTGATATAAATAAATAAATAAA

Lx8 ------------------------------------------------------------

L1MC4 TAAATGGGGGAGAAGGGAAAGCTCTTCCTTACAGTAGAATGCCAACTAATAAATGTAGAA

Lx8 ------------------------------------------------------------

L1MC4 GGAATGATGGAATTAGAAAATCACCATTTGGCAACCATCATAGTAATAATTGATTCAGGC

Lx8 ------------------------------------------------------------

L1MC4 AAGAATCATCAATGGATGCTAAAACTAGTGGGTGAAAGTTTGATGAGNAACAGGATATTT

Lx8 ------------------------------------------------------------

L1MC4 ACATAGTCTCAAAGTATCTCCCCACAAAATACTTATTAATTACAAAGGGGAAAATAGTAA

Lx8 ------------------------------------------------------------

L1MC4 CTTTACAGTGGAGAAACCTGGCAGACACCACCTTAACCAAGTGATCAAAGTTAACATCAC

Lx8 ------------------------------------------------------------

L1MC4 CAGTAATGGGACAAATCGACATCATGTGCCTCCTGATATGATGCACTGAGAAGGACACAA

Lx8 ------------------------------------------------------------

L1MC4 CATCACTTCTGTGGTATTCCTGCCAAAAATGCATAACCTGAATCTAATCATGAGGAAACA

Lx8 ------------------------------------------------------------

L1MC4 TCAGACAAACCCAAATTGAGGGACATTCTACAAAATAACTGGCCTGTACTCTTCAAAAAT

Lx8 ------------------------------------------------------------

L1MC4 GTCAAGGTCATGAAAGACAAAGAAAGACTGAGGAACTGTTCCAGATTAAAGGAGACTAAA

Lx8 ------------------------------------------------------------

L1MC4 GAGACATGACAACTAAATGCAACGCGTGATCCTGGATTGGATCCTGGACCAGANTTTTTT

Lx8 ------------------------------------------------------------

L1MC4 TTGCTATAAAGGACATTATTGGGACAACTGGCGAAATTTGAATAAGGTCTGTAGATTAGA

Lx8 ------------------------------------------------------------

L1MC4 TAATAGTATTGTATCAATGTTAATTTCCTGATTTTGATNATTGTACTGTGGTTATGTAAG

Lx8 ------------------------------------------------------------

L1MC4 AGAATGTCCTTGTTTTTAGGAAATACACACTGAAGTATTTAGGGGTAANGGGGCATCATG

Lx8 ------------------------------------------------------------

L1MC4 TCTGCAACTTACTCTCAAATGGTTCAGAAAAAAAAATATGTATATGNANACAGAGAATGA

Lx8 ------------------------------------------------------------

L1MC4 TAAAGCAAATGTGGCAAAATGTTAACATTTGGGGAATCTGGGTGAAGGGTATACGGGAAT

Lx8 ------------------------------------------------------------

L1MC4 TCTTTGTACTATTCTTGCAACTTTTCTGTAAGTCTGAAATTATTTCAAAATAAAAAGTTA

Lx8 -----

L1MC4 AAAAA
