# Supplementary material for: Comparative analysis of transposed element insertion within human and mouse genomes reveals Alu's unique role in shaping the human transcriptome
Source: Genome Biol. 2007 Jun 27;8(6):R127. doi: 10.1186/gb-2007-8-6-r127 (PMC2394776; doi:10.1186/gb-2007-8-6-r127)
Supplement: Additional data file 7 — Presented is an illustration showing the populations of different families of L1 within human and mouse. [file gb-2007-8-6-r127-S7.doc]

**Figure S3:** **L1 has different intronic and exonic family populations between human and mouse**

The different L1 subfamily population within human and mouse:

**Mouse L1 intronic population:**

**Human L1 intronic population:**
