# Supplementary material for: Comparative analysis of transposed element insertion within human and mouse genomes reveals Alu's unique role in shaping the human transcriptome
Source: Genome Biol. 2007 Jun 27;8(6):R127. doi: 10.1186/gb-2007-8-6-r127 (PMC2394776; doi:10.1186/gb-2007-8-6-r127)
Supplement: Additional data file 8 — Presented is a table of cDNA and EST accessions, confirming the noncanonical 3' splice site of the alternative intron within CWF19L1 gene. [file gb-2007-8-6-r127-S8.doc]

**Table S5: cDNA and EST accession confirming the non-canonical 3' splice site that we observed in the alternative intron within the last exon of CWF19L1 gene**

**A - cDNA/EST accessions confirming the non-canonical spliced intron (Fig3A, iv)**

| **Origin** | **accession** |
| --- | --- |
| GenBank | AK023984 |
|  | BC008746 |
|  | AK225590 |
|  | AK001860 |
| ESTs | AI767774 |
|  | BU679686 |
|  | AI215794 |
|  | AI681259 |
|  | AI553754 |
|  | BX351112 |
|  | AA865287 |
|  | AI340302 |

**B – cDNA/EST accessions confirming the un-spliced intron (Fig3A, ii)**

| **Origin** | **accession** |
| --- | --- |
| GenBank | AL832515 |
|  | AK097895 |
|  | AK055313 |
|  | AK000876 |
