# Supplementary material for: Comparative analysis of transposed element insertion within human and mouse genomes reveals Alu's unique role in shaping the human transcriptome
Source: Genome Biol. 2007 Jun 27;8(6):R127. doi: 10.1186/gb-2007-8-6-r127 (PMC2394776; doi:10.1186/gb-2007-8-6-r127)
Supplement: Additional data file 9 — Presented is a table showing the domains contributed by TE exons. [file gb-2007-8-6-r127-S9.doc]

**Table S6:** **Domain contribution:**

| **RE** | **Domain name** | **Number of TE exonizations containing the domain** |
| --- | --- | --- |
| Alu | Casein kinase II phosphorylation site | 23 |
| Alu | N-myristoylation site | 29 |
| Alu | Protein kinase C phosphorylation site | 16 |
| Alu | c-AMP and cGMP dependent protein kinase phosphorylation site | 6 |
| Alu | N-glycosylation site | 3 |
| Alu | Microbodies C-terminal targeting signal | 1 |
| MIR | Casein kinase II phosphorylation site | 3 |
| MIR | Protein kinase C phosphorylation site | 1 |
| L1 | Protein kinase C phosphorylation site | 5 |
| L1 | Prenyl group binding site (CAAX box) | 1 |
| L1 | Casein kinase II phosphorylation site | 1 |
| L1 | N-myristoylation site | 2 |
| L1 | N-glycosylation site | 1 |
| L2 | N-myristoylation site | 1 |
| L2 | Protein kinase C phosphorylation site | 3 |
| L2 | Casein kinase II phosphorylation site | 1 |
