# Supplementary material for: Comparative analysis of transposed element insertion within human and mouse genomes reveals Alu's unique role in shaping the human transcriptome
Source: Genome Biol. 2007 Jun 27;8(6):R127. doi: 10.1186/gb-2007-8-6-r127 (PMC2394776; doi:10.1186/gb-2007-8-6-r127)
Supplement: Additional data file 10 — Presented is a table of tissue and cancer-specific TEs. [file gb-2007-8-6-r127-S10.doc]

**Table S7: Potential involvement of TE exonizations in cancer and tissue specificity transcripts.** (**A**) Six TE exonizations showing a potential cancer specificity pattern are shown. From left to right: the gene name in which the exonization occurred, the TE family name, the position within the mRNA, the name of the cancerous tissue in which the exon was found, the number of ESTs/mRNAs skipping the exon in cancerous tissue, the number of ESTs/mRNAs containing the exon in cancerous tissue, the number of ESTs/mRNAs skipping the exon in normal tissue, the number of ESTs/mRNAs containing the exon in normal tissue and the LOD score. (**B** and **C**) Potentially tissue specific (TS) exons in the human and mouse transcriptomes, respectively. From left to right: the gene name in which the exonization occurred, the TE family name, the position within the mRNA, the name of the tissue specificity of the exon, the number of ESTs/mRNAs skipping the exon within the tissue, the number of ESTs/mRNAs containing the exon within the tissue, the number of ESTs/mRNAs skipping the exon in all other tissues, the number of ESTs/mRNAs containing the exon in all other tissues and the TS score.

A

| **Gene** | **RE** | **Position** | **Tissue** | **C1** | **C2** | **N1** | **N2** | **LOD** |
| --- | --- | --- | --- | --- | --- | --- | --- | --- |
| ACAD9  [NM_014049](http://www.ncbi.nlm.nih.gov/entrez/query.fcgi?cmd=Search&db=Nucleotide&term=NM_014049&doptcmdl=GenBank&tool=genome.ucsc.edu) | AluJb | CDS | Adenocarcinoma | 29 | 6 | 33 | 0 | 2.14 |
| YY1 associated protein 1  [NM_139118](http://genome-www5.stanford.edu/cgi-bin/SMD/source/sourceResult?option=Number&criteria=NM_139118&choice=Gene) | AluJb | CDS | Rhabdomyosarcoma  Neuroblastoma | 63 | 7 | 68 | 0 | 2.43 |
| AMP activated protein kinase, non-catalytic  [NM_017431](http://genome-www5.stanford.edu/cgi-bin/SMD/source/sourceResult?option=Number&criteria=NM_017431&choice=Gene) | AluJo | CDS | Neuroblastoma  Epitheloid carcinoma  Large cell carcinoma | 65 | 4 | 118 | 0 | 2.18 |
| Nel like 1 precursor  [NM_006157](http://genome-www5.stanford.edu/cgi-bin/SMD/source/sourceResult?option=Number&criteria=NM_006157&choice=Gene) | AluSx | CDS | Rhabdomyosarcoma | 0 | 2 | 17 | 0 | 3.12 |
| Ku70 binding protein 3  [NM_033276](http://genome-www5.stanford.edu/cgi-bin/SMD/source/sourceResult?option=Number&criteria=NM_033276&choice=Gene) | AluJo | CDS | Amelanotic melanoma | 6 | 4 | 16 | 0 | 2.31 |
| Active breakpoint cluster region related  [NM_021962](http://genome-www5.stanford.edu/cgi-bin/SMD/source/sourceResult?option=Number&criteria=NM_021962&choice=Gene) | AluY | CDS | Large cell carcinoma | 5 | 2 | 41 | 0 | 2.53 |

B human

| **Gene** | **RE** | **Position** | **Tissue** | **T1** | **T2** | **W1** | **W2** | **TS** |
| --- | --- | --- | --- | --- | --- | --- | --- | --- |
| Sentrin/SUMO-specific protease 7  NM_020654 | L1 | CDS | brain | 8 | 0 | 2 | 25 | 99.8 |
| Synaptotagmin I  NM_005639 | L1 | CDS | placenta | 4 | 0 | 2 | 20 | 96.8 |
| Tissue inhibitor of metalloproteinase 2  NM_003255 | CR1 | Alt. CDS | testis | 16 | 1 | 1 | 24 | 99.9 |
| Transmembrane and ubiquitine-like domain (TMUB2)  NM_177441 | MIR | UTR | nerve | 8 | 2 | 32 | 66 | 96.7 |
| Translocase of inner mitochondrial membrane 8  NM_012459 | MIR | UTR | lung | 11 | 4 | 73 | 124 | 96.1 |
| p53 induced protein  NM_006034 | MIR | UTR | testis | 5 | 0 | 1 | 31 | 98.4 |
| Suppressor of G2 allel of SKP1  NM_006704 | Alu | CDS | muscle | 4 | 0 | 40 | 71 | 96.7 |
| Hypothetical protein LOC55020  NM_017931 | Alu | CDS | placenta | 4 | 0 | 3 | 37 | 96.8 |
| Jak and microtubule interacting protein1 [NM_014790](http://genome-www5.stanford.edu/cgi-bin/SMD/source/sourceResult?option=Number&criteria=NM_014790&choice=Gene) | Alu | CDS | embryo | 4 | 0 | 3 | 15 | 96.6 |
| Adaptor-related protein complex 3, mu 1 subunit  NM_133593 | Alu | UTR | eye | 0 | 6 | 41 | 23 | 97.9 |
| GRB2-associated binding protein 1  NM_002039 | Alu | CDS | brain | 13 | 3 | 1 | 6 | 95.8 |
| Hypothetical protein LOC54932  NM_017820 | Alu | CDS | prostate | 4 | 0 | 1 | 19 | 96.8 |
| DnaJ (Hsp40) homolog, superfamily B, member 7  NM_145174 | Alu | UTR | skin | 7 | 0 | 14 | 31 | 99.0 |
| Mitochondrial ATP-Mg/Pi carrier protein  NM_024103 | Alu | UTR | eye | 4 | 0 | 5 | 18 | 96.5 |
| zinc finger MYM domain containing 1  NM_024772 | Alu | UTR | brain | 4 | 0 | 3 | 11 | 95.1 |
| Zinc finger protein 789  NM_213603 | MER2 | CDS | testis | 4 | 0 | 8 | 22 | 96.34 |
| Phosphoinositide-specific phospholipase C beta 1  NM_015192 | MER1 | CDS | ovary | 4 | 0 | 1 | 14 | 96.85 |
| Thiamin pyrophosphokinase 1  NM_022445 | ERV1 | CDS | testis | 4 | 0 | 2 | 21 | 96.87 |
| Amylase, alpha 1A;  salivary precursor  NM_004038 | ERV1 | UTR | mouth/oral | 13 | 0 | 1 | 18 | 99.99 |

C mouse

| **Gene** | **RE** | **Position** | **Tissue** | **T1** | **T2** | **W1** | **W2** | **TS** |
| --- | --- | --- | --- | --- | --- | --- | --- | --- |
| Probable ubiquitine carboxyl-terminal hydrolase CYLD  NM_173369 | L1 | UTR | heart | 4 | 0 | 39 | 136 | 96.8 |
| ATP-binding cassette, subfamily C, member 5  NM_176839 | B1 | CDS | kidney | 4 | 0 | 1 | 51 | 96.8 |
| Sec 13-like protein  NM_028112 | B4 | CDS | genitourinary tract | 5 | 0 | 3 | 37 | 98.4 |
| ST6  NM_011373 | MIR | Alt. CDS | intestine | 3 | 14 | 41 | 14 | 99.6 |
