# Supplementary material for: Comparative analysis of transposed element insertion within human and mouse genomes reveals Alu's unique role in shaping the human transcriptome
Source: Genome Biol. 2007 Jun 27;8(6):R127. doi: 10.1186/gb-2007-8-6-r127 (PMC2394776; doi:10.1186/gb-2007-8-6-r127)
Supplement: Additional data file 11 — Presented is a table of the potential splice sites of all TEs. [file gb-2007-8-6-r127-S11.doc]

**Table S8: potential splice sites**

| **TE family/TE class** | **sense/sntisense** | **Splice site** | **number** |
| --- | --- | --- | --- |
| L2/L2 | sense | 3'ss | 18 |
|  |  | 5'ss | 9 |
|  | antisense | 3'ss | 4 |
|  |  | 5'ss | 90 |
| L3/CR1/LINE | sense | 3'ss | 7 |
|  |  | 5'ss | 40 |
|  | antisense | 3'ss | 26 |
|  |  | 5'ss | 27 |
| MIR/MIR/SINE | sense | 3'ss | 0 |
|  |  | 5'ss | 8 |
|  | antisense | 3'ss | 0 |
|  |  | 5'ss | 20 |
| AluSx/Alu/SINE | sense | 3'ss | 0 |
|  |  | 5'ss | 4 |
|  | antisense | 3'ss | 2 |
|  |  | 5'ss | 4 |
| B1_mus1/B1/SINE | sense | 3'ss | 0 |
|  |  | 5'ss | 4 |
|  | antisense | 3'ss | 2 |
|  |  | 5'ss | 3 |
| B3/B2/SINE | sense | 3'ss | 1 |
|  |  | 5'ss | 3 |
|  | antisense | 3'ss | 2 |
|  |  | 5'ss | 11 |
| B4/B4/SINE | sense | 3'ss | 0 |
|  |  | 5'ss | 6 |
|  | antisense | 3'ss | 2 |
|  |  | 5'ss | 7 |
| ID4/ID/SINE | sense | 3'ss | 0 |
|  |  | 5'ss | 5 |
|  | antisense | 3'ss | 0 |
|  |  | 5'ss | 0 |
| Lx8/L1/LINE | sense | 3'ss | 4 |
|  |  | 5'ss | 6 |
|  | antisense | 3'ss | 6 |
|  |  | 5'ss | 18 |
| L1MC4/L1/LINE | sense | 3'ss | 6 |
|  |  | 5'ss | 22 |
|  | antisense | 3'ss | 9 |
|  |  | 5'ss | 32 |
| MLT1A/MaLR/LTR | sense | 3'ss | 1 |
|  |  | 5'ss | 5 |
|  | antisense | 3'ss | 3 |
|  |  | 5'ss | 3 |
| Charlie1/MER1_type/DNA | sense | 3'ss | 6 |
|  |  | 5'ss | 30 |
|  | antisense | 3'ss | 12 |
|  |  | 5'ss | 33 |
