# Supplementary material for: Comparative analysis of transposed element insertion within human and mouse genomes reveals Alu's unique role in shaping the human transcriptome
Source: Genome Biol. 2007 Jun 27;8(6):R127. doi: 10.1186/gb-2007-8-6-r127 (PMC2394776; doi:10.1186/gb-2007-8-6-r127)
Supplement: Additional data file 12 — Presented is an illustration showing RT-PCR of ACAD9 Alu exonization in different human cell lines. [file gb-2007-8-6-r127-S12.doc]

**Figure S4: RT-PCR ACAD9 gene in different human cell lines.** Total RNA was extracted from 293T human kidney adenocarcinoma cell lines (lane 1), DU-145 prostate carcinoma (lane 2), PC-3 prostate adenocarcinoma (lane 3), MCF-7 human breast adenocarcinoma (lane 4), HT1080 human fibrosarcoma (lane 5), stem-cells (bone marrow) (lane 6), 393RM human lymphoblast (lane 7), ES-2 Human clear cell carcinoma of ovary-fibroblast (lane 8), SK-OV-3 human epithelial ovarian adenocarcinoma (lane 9); cDNA synthesis was performed using primers that were designed on the flanking exons of the intronic Alu**.**

**
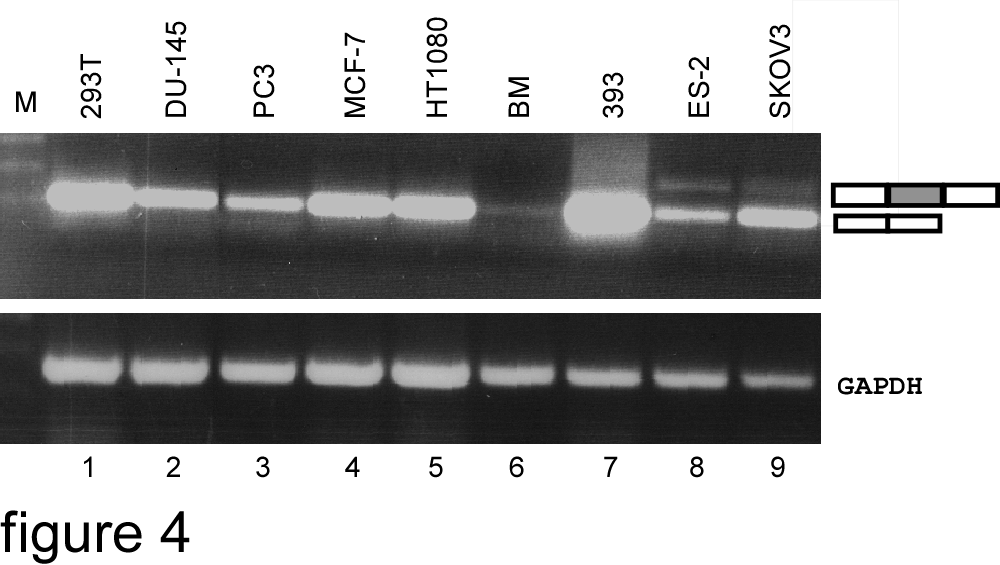
**
